# A Novel Function for Lysyl Oxidase in Pluripotent Mesenchymal Cell Proliferation and Relevance to Inflammation-Associated Osteopenia

**DOI:** 10.1371/journal.pone.0100669

**Published:** 2014-06-27

**Authors:** Roozbeh Khosravi, Katharine L. Sodek, Wan-Peng Xu, Manish V. Bais, Debashree Saxena, Michael Faibish, Philip C. Trackman

**Affiliations:** Department of Molecular and Cell Biology, Boston University Henry M. Goldman School of Dental Medicine, Boston, Massachusetts, United States of America; University of Maryland, United States of America

## Abstract

Lysyl oxidase is a multifunctional enzyme required for collagen biosynthesis. Various growth factors regulate lysyl oxidase during osteoblast differentiation, subject to modulation by cytokines such as TNF-α in inflammatory osteopenic disorders including diabetic bone disease. Canonical Wnt signaling promotes osteoblast development. Here we investigated the effect of Wnt3a and TNF-α on lysyl oxidase expression in pluripotent C3H10T1/2 cells, bone marrow stromal cells, and committed osteoblasts. Lysyl oxidase was up-regulated by a transcriptional mechanism 3-fold in C3H10T1/2 cells, and 2.5-fold in bone marrow stromal cells. A putative functional TCF/LEF element was identified in the lysyl oxidase promoter. Interestingly, lysyl oxidase was not up-regulated in committed primary rat calvarial- or MC3T3-E1 osteoblasts. TNF-α down-regulated lysyl oxidase both in Wnt3a-treated and in non-treated C3H10T1/2 cells by a post-transcriptional mechanism mediated by miR203. Non-differentiated cells do not produce a collagen matrix; thus, a novel biological role for lysyl oxidase in pluripotent cells was investigated. Lysyl oxidase shRNAs effectively silenced lysyl oxidase expression, and suppressed the growth of C3H10T1/2 cells by 50%, and blocked osteoblast differentiation. We propose that interference with lysyl oxidase expression under excess inflammatory conditions such as those that occur in diabetes, osteoporosis, or rheumatoid arthritis can result in a diminished pool of pluripotent cells which ultimately contributes to osteopenia.

## Introduction

Ostepenia can be caused by a variety of systemic conditions among which are osteoporosis, rheumatoid osteoarthritis and diabetes [1]. Diabetic osteopenia leads to elevated incidences of foot fractures, and poor bone healing after orthopedic and dental procedures. Diabetic osteopenia is characterized by reduced osteoblast bone synthetic activity, while osteoporosis and osteoarthritis are characterized by a greater proportion of bone resorption [1], [2]. Diabetic bone contains deficient levels of normal biosynthetic lysyl oxidase-derived cross-links [3], [4], and increased levels of advanced glycation end product modification [2], [5]. Elevated levels of inflammation occur in virtually all osteopenic diseases [6]–[8].

The canonical Wnt pathway contributes to bone formation and activates β-catenin-dependent transcription. Wnt signaling is essential for pre-osteoblast differentiation and mineralized tissue homeostasis and induces the proliferation of pluripotent cells and pre-osteoblasts; as well as the survival of osteoblasts and osteocytes [9]. The canonical Wnt signaling pathway is mediated by the frizzled receptors and low-density lipoprotein receptor-related protein (LRP5/6) co-receptors, culminating in the nuclear accumulation of β-catenin and its co-activation of TCF/LEF transcription factors [10]. A mutation in the Wnt co-receptor LRP5 leads to diminished Wnt-signaling and reduced bone mass in osteoporosis-pseudoglioma syndrome (OPPG) [11].

Inflammation, reactive oxygen species (ROS) and TNF-α levels are elevated in diabetes and enhance FOXO1/β-catenin interactions at the expense of TCF/LEF-dependent transcription [12]–[14]. This mechanism reduces osteogenic TCF/LEF signaling, promotes pathways that lead to increased apoptosis, and can interfere with bone cell differentiation and bone formation [15].

Wnt3a was reported to up-regulate lysyl oxidase in C3H10T1/2 cells, a model of pluripotent mesenchymal progenitor cells [16], though the mechanism and significance of this finding was not investigated. Lysyl oxidase is critically important for collagen maturation, collagen structure and bone strength [17], [18]. C3H10T1/2 cells can be directed toward adipocyte, chondrocyte or osteoblast phenotypes [19]–[21]. Here we investigate the hypothesis that Wnt3a transcriptional up-regulation of lysyl oxidase could contribute to differentiation of C3H10T1/2 cells toward a chondrocyte or osteoblast phenotype and that Wnt3a would stimulate lysyl oxidase expression in committed osteoblasts in light of the known activity of lysyl oxidase in bone collagen biosynthesis and maturation. In addition, we evaluated whether TNF-α could inhibit Wnt3a up-regulation of lysyl oxidase by interfering with Wnt3a-stimulated transcription of lysyl oxidase.

Findings in C3H10T1/2 cells and in primary bone marrow stromal cells revealed that lysyl oxidase is up-regulated by Wnt3a as expected and TNF-α attenuated lysyl oxidase mRNA levels. Wnt3a, however, did not up-regulate lysyl oxidase in MC3T3-E1 cells or in primary rat calvaria-derived osteoblasts. TNF-α down-regulated lysyl oxidase at the post-transcriptional level in C3H10T1/2 cells by reducing the half-life of lysyl oxidase mRNA mediated by miR203, and not by inhibition of lysyl oxidase transcription as originally predicted. These pluripotent cells are non-differentiated and do not make a significant collagenous extracellular matrix, raising the question regarding the biological function of lysyl oxidase in non-differentiated cells. Findings demonstrate a strong dependence of these cells on lysyl oxidase for proliferation. Thus, data identify a novel activity of lysyl oxidase which fosters pluripotent cell proliferation. We propose that down-regulation of lysyl oxidase in pluripotent cells by TNF-α in inflammatory diseases can lead to a smaller pool of precursor cells ultimately leading to a diminished population of bone or cartilage producing cells, and consequent osteopenia.

## Materials and Methods

### Isolation of primary mouse bone marrow stromal cells (BMSCs) and primary calvarial rat osteoblasts

The Boston University Institutional Animal Care and Use Committee (IACUC) approved both the mouse and rat animal protocols employed. All animal work was performed in accordance with NIH requirements. Primary BMSCs were harvested from the tibia and femur of C57BL/6J mice (JAX cat#00664). 8-Week old mice were euthanized and Minimal Essential Medium (α-MEM) was flushed through the medullary cavities of the femur and tibia using a 27½ gauge needle and cultured in α-MEM at an initial density of 1.5×10^7^ cells/well (6-well plates). Primary rat calvarial osteoblasts were isolated from 16–18 day embryonic CD IGS 001 rats (Charles River Laboratory) as previously described [22].

### Cell culture

MC3T3-E1 cells (cat#CRL-2593) and C3H10T1/2 cells (cat#CCL-226) were purchased from ATCC. Primary rat calvarial osteoblasts, MC3T3-E1 and BMSCs were grown in α-MEM (Invitrogen cat#12571-063) and C3H10T1/2 cells were grown in Eagle's MEM (ATCC cat#30-2003). Media were supplemented with 0.1 mM nonessential amino acids, 10% fetal bovine serum, 100 Units/ml penicillin and 0.1 mg/ml streptomycin, grown at 37°C and 5% CO_2_ in a fully humidified incubator. When cells reached 80% visual confluence cells were re-fed with serum-free medium containing 0.1% bovine serum albumin (BSA) for a minimum of 18 hours prior to initiating experiments.

### Preparation of Wnt-conditioned medium

L-cells which express recombinant Wnt3a (Wnt3a L-cells) and control L-cells which do not express Wnt3a were purchased from ATCC (cat#CRL-2647 and cat#CRL-2648, respectively). Cells were grown in ATCC-Formulated Dulbecco's Modified Eagle's Medium (ATCC cat#302002) supplemented as described above and with 0.4 mg/ml G-418 for Wnt3a L-cells. Wnt3a L-cells and control L-cells were grown to full confluence, re-fed with fresh media without G-418, and media were conditioned for 2 days, and repeated once. Media were pooled, filtered, and aliquots stored at −20°C. Wnt3a- or control-conditioned media at a concentration of 20% in growth medium were used in all experiments. Selected experiments were performed with 150 ng/ml mouse recombinant Wnt3a purchased from Preprotech (cat#315-20).

### Real time PCR

Total RNA was extracted from cells using a Mini RNeasy kit (Qiagen cat#74106). cDNA was synthesized from 2 µg total RNA, using TaqMan Reverse Transcription Reagents (Applied Biosystems cat#N8080234). cDNAs were then subjected to real time PCR analysis to measure selected mRNA levels. The following TaqMan probes were purchased from Applied Biosystems: mouse lysyl oxidase (cat#Mm00495386_m1), 18S rRNA (cat#Hs03003631_g1), mouse lysyl oxidase-like 1 (LOXL1) (cat#Mm001145738_m1), alkaline phosphatase (cat#Mm00475831_m1), beta-2 microglobulin (cat#Mm00437762_m1), glyceraldehyde-3-phosphate dehydrogenase (GAPDH) (cat#Mm99999915_g1), and hypoxanthine phosphoribosyl transferase (HPRT1) (cat#Mm00446968_m1).

### Site-directed mutagenesis of the lysyl oxidase promoter

The 2.5 kbp mouse lysyl oxidase promoter DNA extending from −2,073 bp upstream from the translation start site to position +434 bp, was kindly provided by Dr. Pascal Sommer, Institute of Biology and Chemistry of Proteins, University Claude Bernard, Lyon, France and was re-cloned into the pGL4.10 luciferase reporter construct (Promega cat# E6651) [23], [24] that we here designate as pLOXFFL. Site directed mutagenesis was performed using the QuickChange II XL Site-Direct Mutagenesis Kit (Genomics Agilent cat#200521) to mutate three TCF/LEF conserved consensus sequences [25] within the lysyl oxidase promoter as per the manufacturer's instructions (Table 1). We selected these TCF/LEF binding elements based on in silico analyses of putative element locations and conservation between species using PROMO v.3 and TRANSFAC 7.0 software available online (http://alggen.lsi.upc.es/cgi-bin/promo_v3/promo/promoinit.cgi?dirDB=TF_8.3 and http://www.gene-regulation.com/pub/databases.html#transfac, respectively). Primers were designed using the QuickChange Primer Design Program (www.agilent.com/genomics/qcpd) (Table 2). The three sites that were mutated are as follow: Site#1 was from −913 to −906 upstream of the start codon; site#2 was from −1321 to −1328 upstream of the start codon; site#3 was from −1392 to −1385 upstream of the start codon (Table 3).

**Table 1:** Lysyl Oxidase Promoter Sequence 2473

−2473	CCTCCATAGC	AGATGTCTTC	CCCCAGAAAC	CTGTGGTCAC	GTAATAACCA	TCCCAGCCAT
−2413	GCCCTTCTGG	AAGATAAACT	GCTCATGGGC	ATATGTGCAG	CCTATTTCAG	ATGAGAGATA
−2353	ATTATAACCA	ATCTCTTCCT	GTTTCTCCTT	ATAATTTCTT	TTGTCTATCC	AAATTATATA
−2293	CGTGGTGTTT	TTTTTTTCTT	TCTTCTTTTT	AACTCTATCT	CCTTTACCAG	GTCTAGAGAA
−2233	ACCCAGTAGT	TAATTGTAAG	CCTGGTAGAT	ATCCAGTAAG	ATGACAACTA	GTGTTACCTT
−2173	AGACATAGCT	TTTTTCAGAA	AAGAGTTCAC	TGTGGACTTG	CATGAGGGAA	TTTGTTCTTA
−2113	AAGTTTATCT	TCATCATATA	AGCAATAGAT	TTGCCACATA	TGGACAAAAG	TCGGTGTCAA
−2053	GGATGTAGGT	GCTCAAGGGC	AGGCTCCATT	GAACTTGACT	CCAAAAGCCA	GGCTCCTTAC
−1993	CCTGTCACGT	GATTTACCTA	GCTGAGAGAA	GCAGCCCCAC	CCTCTATGTC	AAACAGGATT
−1933	CTTCCTTGGC	AGAAGACAGA	AACTGACAAT	ATTAATCCAC	AAAAGAATGC	ACTAGAAAGT
−1873	CTGAAGGAAA	AAGCTAAAGA	CTCAAGACTT	TTCCTAGCAG	TGGGAGGAGG	AACGTTTTTG
−1813	ACCATGTGAC	CACCTTAACA	TTCAGATTCT	AGGATGATAG	TTTAATTAAG	TCTCATGACC
−1753	AGTTCCTGAA	TTGAAGAAAT	CCCATTAACG	CCTCGTCAGT	CTAACCACAC	TACTAGGGGT
−1693	CGGGGGTTCC	AGGTAGAATT	AAACAAAACA	AAAAAACAAA	AACAAAACAA	AAGAGAGAGA
−1633	GAGAGAGAGA	GAGAGAGAGA	GAGAGAGAGA	GAGAGAGAAA	AGGGAAAAGG	ATAGGAAGCA
−1573	ATCAAACCAG	CAGATGTCCA	CCACTCTACC	TCATGGGGTG	ATCAGTTATG	GAAAGACAGA
−1513	GGGGAAGAGG	TGAATATTAA	GTGTGTCTAG	ACCACAGCTC	AATGATGGGA	CCAATAAAAT
−1453	AGGTACTAAA	GTCTGGCACT	GCCTAGTAGT	TCAAATCAGA	CCACTTAGTA	CTTTTTTTTT
−1393	T**CTTTGAA**TT	TGGTCCAGAA	CTTCCCAGAC	CCTGAGTAGG	ATGTGAACAG	CTGCTCTATT
−1333	AAGCATCTCT	CTCCAGTTGC	AGACCATCCT	CCTACTCAGC	CTCCTGGCCA	CACCCACTGC
−1273	CTTCTTTGTA	CTAAACTCTG	AACTCCAAGG	CGTTTTCTCT	TGCGACAC**CT**	**TTGTT**TAGAT
−1213	TGGAAGATGA	ATTAAAAAGC	TGCCGAATGC	GTTATTGAAT	TTCAATATTT	AACATCGGTA
−1153	ATTTGATTTT	AAAATGTAAA	GCCAAACCTC	CTTATTAGGC	AAAAAGAAAA	AGCAAGAAGT
−1093	CAGATAAACG	GTCTACTTGA	AGGAGCCTCT	GAGTACTGCC	TTGCCCTTCT	GGCTCTCTGG
−1033	TACACAGTAG	GGATGAAAGA	ATTCTTGTCA	CTTAAAGTGC	AAATTGGGCT	GGTTCTTTTA
−973	AGAACAACAA	CAACAAAAAG	AAAAAAGAGT	CGGATTTATT	CACTTTTTCA	ACGTAGCAAG
−913	**CTTTGTT**CCC	TATAAATTTT	CACTTTGGTT	ATTAAAATAT	TCACTGTAGG	AAACAGATTT
−853	GTAACCCATT	TCTCATATTA	CCTACAGCCA	GAAAAACAAA	ATTTGATATC	CTGGGGTTTA
−793	TTCGCTGAGG	GCGCTTCCCA	TAAAAGTGGG	GTGAGTGTGT	ATTGGGAAAT	TTGTCTGCTT
−733	AACTCCTTTA	AGCATAAGCC	TTAGTCACAA	CCTCCCCCAT	CCCAGAGCAC	ACAGTTTGGC
−673	CCCCACCACT	CTCCCCACCT	CCTGCTTCCC	GCCTCTCCAG	GGTTGGTGAC	CTAATAGCAT
−613	TTTTCTTCAT	GCATATTTTG	GCTTGGGCCC	ATGGCCTGGC	TGCCTTCGTC	TGTCTGAGTC
−553	TTTTGAAATT	CCTGCATGTT	CGGCCCAGAT	TAAGTCGAGT	GTGTCTCAGG	ATGTGTGTTC
−493	CGTTTTGTTC	TTTCCCCTTA	ACTCTCCCTG	TGCAACGTGT	CTGGGGAGGA	GGGGAGGGGG
−433	GCGGGGAGGG	GAGGGAGGGG	CAGCGTGGAG	GAGCTGTCCG	CCTTGCACGT	TTCCAATCAC
−373	ATTACGTGAA	CAAATAGCGG	AGGGGCAGCG	GGGCCAGAAC	GGCTTGTGTA	ACTGCAAACT
−313	GGTCAGAAAG	TTTAAAATCT	CTCCTCCTCC	TTCTACTCCA	GACTCTGTGC	GCTTTCCCAG
−253	ACCTTCGTGC	GCAGCTCCCC	GTCGCCTTCC	AGGGCTGGGA	AAGGGGAGAG	GAGGACCGTG
−193	CCACGTCCGA	CGGCAGCCTG	GGCTGGGGGC	AGGGTCTGCT	GTTCGCCCTG	GCACGACTCC
−133	CTGCGACCCA	TCCCCGCGCC	TCGCCCTTCT	CCTCCCTGCT	CGGAGAGGTC	TCCCTCCTTC
−73	GTGGGATCTG	AGTCCCGGTC	TTCCTTTTTC	TCCTAGCCAC	GTCCTCCCCG	AGAAGGGACG
−13	AGCCGGGAGC	ACC***ATG***CGTT	TCGCCTGGGC	TGTGCTCCTT	CTGGGGCCAC	TGCAGCTTTG
48	TCCCCTTCTC	CGCTGCGCCC	CG			

The translation start site is in bold italics. Putative TCF/LEF elements are underlined in bold face font.

**Table 2:** Primers for Site-directed Mutagenesis of the Lysyl Oxidase Promoter.

Mutated site	Primer sequence
Site #1	Sense: 5′-tattcactttttcaacgtagcaagctttggcccctataaattttcactttgg-3′
−913 to −906	Antisense: 5′-ccaaagtgaaaatttataggggccaaagcttgctacgttgaaaaagtgaata-3′
Site #2	Sense: 5′-gcgttttctcttgcgacacctttggctagattggaagatgaattaaaaag-3′
−1321 to −1328	Antisense: 5′-ctttttaattcatcttccaatctagccaaaggtgtcgcaagagaaaacgc-3′
Site #3	Sense: 5′-gaccacttagtacttttttttttctttggctttggtccagaacttcccagac-3′
−1392 to −1385	Antisense: 5′-gtctgggaagttctggaccaaagccaaagaaaaaaaaaagtactaagtggtc-3′

**Table 3:** Wild-type and Mutant Sequences of TCF/LEF Elements in the Lysyl Oxidase Promoter.

Mutated site	Wild-type sequence	Mutant sequence
Site #1	AAGCTTTGTTCCC	AAGCTTTG**GC**CCC
−913 to −906		
Site #2	CACCTTTGTTTAG	CACCTTTG**GC**TAG
−1321 to −1328		
Site #3	TTTCTTTGAATTT	TTTCTTTG**GC**TTT
−1392 to −1385		

**TCF/LEF consensus sequence: CTTTG(A/T)(A/T).**

### Transfection

FuGene-6 reagent (Roche# 11914443001) was used to transiently transfect cells with various constructs: (i) lysyl oxidase promoter firefly-luciferase construct (pLOXFFL); (ii) TCF/LEF mutated lysyl oxidase promoter firefly-luciferase constructs (pmLOXFFL); (iii) pCS2+DKK1-flag (plasmid 16690) and SOST/pcDNA3.1+ (plasmid 10842) both from Addgene; (iv) miR203 RenSP-luciferase construct (miR203-RenSPL); (v) R01 control RenSP-luciferase construct (R01-RenSPL); (vi) Renilla luciferase thymidine kinase (pRL-TK, Promega cat#E2241). DNA:FuGene-6 ratio was 1∶3 (0.33 µg DNA/1 µl FuGene-6 per well of a 24-well plate to a final volume of 300 µl per well). Cells were incubated with the DNA:FuGene-6 mixture and in serum-and antibiotic-free media for 6 hr. Cells were then fed with fresh medium, and after 24 hours, transfected cells medium was replaced with serum-free medium containing 0.1% BSA.

C3H10T1/2 cells were transfected with miR203 mimic (Syn-mmu-miR-203 miScript microRNA Mimic, Qiagen cat#MSY0000236) or a control microRNA mimic (AllStars Negative Control siRNA, Qiagen cat#1027280) using Hi-Perfect (Qiagen cat#301705). Cells were seeded in a 6-well plate at the density of 1×10^6^ per well, and the next day, were incubated with 2 ml per well of transfection master mix [Eagle's MEM supplemented with 5 nM siRNAs (miR203 mimic or negative control mimic), 20 µl Hi-perfect reagent, and 0.1% BSA] for 8 hours. The medium was refreshed every second day. Total RNA was isolated from cell layers on day 5 post-transfection, and subjected to real time PCR to measure lysyl oxidase mRNA levels.

### microRNA PCR array

For microRNA analysis, total RNA, including microRNA, was extracted from cell lysates using a miRNeasy Mini Kit (Qiagen cat#217004). Next, the RT^2^ microRNA First Strand Kit (Qiagen cat# 331401) was used to synthesize cDNA from 4 µg of total RNA. The microRNA PCR array (Mouse miRNome RT^2^ Whole Genome microRNA PCR Array, Qiagen cat#MAM-200A-2) was used to probe for 440 mouse microRNAs normalized to the average levels of the housekeeping small nuclear RNAs, SNORD 68, 66 and Rnu6.

### Luciferase assay

To examine lysyl oxidase promoter activity, C3H10T1/2 cells at 80% visual confluence were transfected with the lysyl oxidase promoter reporter construct (pLOXFFL) and a thymidine kinase renilla luciferase reporter (pRL-TK) construct (9∶1 ratio, respectively). 24 hours post-transfection, cells were serum-starved (0.1% BSA) overnight and treated with Wnt3a- or control-conditioned medium for 24 hours. Cells were lysed and firefly and renilla luciferase activities were separately measured using a Dual Luciferase Reporter Assay System (Promega cat#E1910). The data were first normalized by calculating the ratio of firefly to renilla luciferase activity then presented as a fold change between Wnt3a treated and control cells.

To functionally assess for TNF-α-induced miR203, 80% visually confluent cells were transfected with miR203 RenSP-luciferase construct (miR203-RenSPL, cat#S880167) or R01 scrambled control RenSP-luciferase construct (R01-RenSPL, cat#S790001), using FuGene-6 as described above. Both constructs were purchased from SwitchGear Genomics. Cells were lysed and the luciferase activity was measured using LightSwitch Luciferase Assay Kit (SwitchGear cat#LS010). The data presented as a ratio of miR203-RenSPL reporter to R01-RenSPL reporter.

### Lentivirus transduction

Two independent lysyl oxidase shRNAs (Sigma Mission TRCN0000011850 and TRCN0000011852) were used to knock down lysyl oxidase in C3H10T1/2 cells. Lentiviral transduction of C3H10T1/2 cells was executed as follows: Human 293T cells were transfected with either lysyl oxidase shRNA plasmids or control shRNA plasmid (target sequence: CCTAAGGTTAAGTCGCCCTCG; Addgene, #1864), in the presence of the packaging plasmids (VSVG and Delta-R 8.2; Addgene), using FuGene 6 (Roche# 11914443001). A firefly luciferase shRNA plasmid was used as the negative control [26]. Media containing viral particles were collected on days 3 and 5 post-transfection. Collected media were pooled and centrifuged at 40,000× g and the viral pellet was suspended in a small volume of PBS. In growth curve experiments, the primary osteoblasts were transduced with lysyl oxidase shRNAs or control shRNA at 80–90% visual confluence for 24 hours. The medium was then refreshed to include 0.4 µg/ml puromycin for 48 hours to select for transduced cells. After two days, transduced cells were seeded as described for each experiment.

### Growth curves

C3H10T1/2 cells transduced with either lysyl oxidase shRNA plasmids or control shRNA plasmid were seeded at a density of 20,000 cells per well in 12-well plates. The attached cells after 8 hours were detached by trypsin-EDTA and were counted using a hemacytometer. The number of cells at this point was designated as Day 0 in order to establish that the number of cells in the control and knock-down groups at the beginning of the experiment was equivalent. Cells in triplicate wells were then counted each day for the remainder of the experiment. The number of cells as a function of post-seeding days were plotted to compare the growth curve of lysyl oxidase knockdown and control cells.

### Short-term proliferation assay

The CyQuant cell proliferation reagent assay (C7026) from Invitrogen, was used to measure the cell proliferation. Freshly transduced and selected cells were seeded at a density of 5000 cells per well in each of eight 24-well plates per experimental group and grown under standard cell culture conditions. On the following day the media were removed from four wells and washed with PBS, and then stored at −80°C (Day 0). After 24 hours media were removed from the remaining four 24-well plates, washed with PBS, and plates stored at −80°C. For measurement, plates were thawed at room temperature for 30 minutes and 200 µl of CyQuant GR dye/cell lysis buffer (Molecular Probes) was added and gently mixed. Samples were incubated in the dark for 5 min at room temperature. Following incubation, 200 µl volumes of cell suspensions, including standards were added to wells of a black fluorescent microliter plate (Thermo Fisher Scientific, UK) and the fluorescence of samples was measured with a TriStar LB 941(Berthold Technologies) with excitation at 420 nm and emission detection at 535 nm.

### Western blot

Freshly transduced C3H10T1/2 cells were grown in 10 cm plates in Eagle's MEM (ATCC cat#30-2003). Cell layers were extracted into sample buffer (0.5 M Tris pH 6.8, glycerol 10% SDS, 5% β-mercaptoethanol) for each condition, boiled for 5 min and stored at −20°C and subjected to Western blotting as we have previously described [27]. Primary antibodies used were affinity-purified anti-rat lysyl oxidase [27], active caspase-3 (Trevigen) and β-actin (Cell Signaling) for normalization. Digital photos from the X-ray films of Western blots were captured using a VersaDoc Imaging System (Biorad). Densitometry analysis was carried using the gel analyzer feature of the Image J software (http://rsbweb.nih.gov/ij/) according to the user manual (IJ version 1.46r, section 30.13). Films with non-saturated exposure were employed for densitometry.

### DNA fragmentation assay

C3H10T1/2 cells freshly transduced with two different LOX shRNAs or control shRNA were seeded at 1×10^6^ cells per 10 cm^2^ plate in Eagle's MEM (ATCC cat#30-2003) supplemented with 0.1 mM nonessential amino acids, 10% fetal bovine serum, 100 Units/ml penicillin and 0.1 mg/ml streptomycin. Five plates per group were grown at 37°C and 5% CO_2_ in a fully humidified incubator. At confluence, cells were then lysed in 0.2% Triton X-100, 10 mM Tris-HCl (pH-7.4), 10 mM EDTA. Samples were centrifuged and supernatants were extracted with phenol∶choloroform∶isoamyl alcohol (25∶24∶1) followed by chloroform∶isoamyl alcohol (24∶1) twice. DNA was precipitated by adding 5 M NaCl to the final concentration of 300 mM and 2.5 volume of ice cold 100% ethanol. Pellets were washed with 70% ethanol, dried and DNA was dissolved in 10 mM of Tris-HCl, (pH 7.5), 1 mM EDTA. DNA samples (25 µg each) were subjected to 2% agarose gel electrophoresis, stained with ethidium bromide, and photographed under UV light.

### Caspase activation assay

C3H10T1/2 cells freshly transduced with two different LOXshRNA lentivirus particles (LOX shRNA1850, and LOX shRNA1852) or with a control shRNA, were grown in 10 cm^2^ plates and at confluence extracted for Western blotting. In addition, a positive control for caspase activation consisted of non-transduced C3H10T1/2 subjected to 20 Gy of ionizing radiation as previously described [28]. The corresponding negative control was non-irradiated cells grown at the same time. Cell layers were extracted into sample buffer (0.5 M Tris pH 6.8, glycerol 10% SDS, 5% β-mercaptoethanol), boiled for 5 min and stored at −20°C and subjected to Western blotting for activated caspase 3.

### C3H10T1/2 cell differentiation assay

For experiments in which C3H10T1/2 cells were cultured under conditions to permit differentiation, media were changed at confluence to α-MEM containing 10% serum, 1% penicillin/streptomycin, 2 µg/ml L-ascorbic acid, 10 nM dexamethasone, and 10 mM β-glycerophosphate. Media were refreshed every second day. Cultures were fixed and stained with Alizarin Red at intervals as we have described previously [22].

### Statistical analyses

Student's t-test at 95% confidence was employed in our analyses. The unequal variance (heteroscedastic) assumption was utilized in the Student's t-test since the standard deviations could potentially vary between groups and were therefore not assumed to be identical. Significance was declared at p<0.05.

## Results

### Wnt3a up-regulates lysyl oxidase in progenitor cells

**Figure 1:**
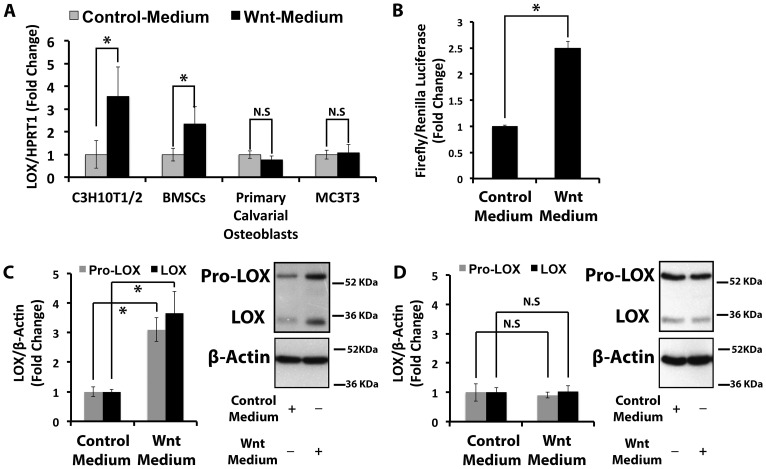
Wnt3a transcriptionally up-regulates lysyl oxidase mRNA levels in C3H10T1/2 pluripotent progenitor cells. Serum-depleted cells were treated with Wnt3a- or control-conditioned medium for 24 hours. Total RNA and protein were extracted and subjected to real time PCR and Western blotting analyses. **A)** The bar graph presents lysyl oxidase mRNA levels in response to Wnt3a in C3H10T1/2 cells (n = 6), mouse primary bone marrow stromal cells (BMSCs, n = 3), rat primary calvarial osteoblasts (n = 9), and a mouse pre-osteoblast cells (MC3T3-E1, n = 3). Data presented for C3H10T1/2 cells, BMSCs and primary calvarial osteoblasts were pooled from two independent experiments; data from MC3T3-E1 cells were from one experiment performed in triplicate. Data shown are means ± SD (*, p<0.05, N.S, not significant; Student's t-test). **B)** The bar graph shows the fold change of lysyl oxidase transcriptional activity in response to Wnt3a in C3H10T1/2 cells (n = 3). Data are from one representative experiment performed in triplicate of three independent experiments, all showing significantly increased lysyl oxidase transcription after Wnt3a treatment. Data shown are means ± SD (*, p<0.05; Student's t-test). **C)** The bar graph presents lysyl oxidase protein levels in response to Wnt3a in C3H10T1/2 cells (n = 3). Data shown are means ± SD (*, p<0.05; Student's t-test). **D)** Lysyl oxidase protein levels in response to Wnt3a in rat primary calvarial cells (n = 6). Data shown are means ± SD N.S, not significant; Student's t-test).

Wnt regulation of lysyl oxidase was assayed in cells representing two stages of differentiation. The C3H10T1/2 cell line and primary bone marrow stromal cells (BMSCs) are pluripotent mesenchymal progenitor cell models; whereas the MC3T3 cell line and primary rat calvarial osteoblasts are committed osteoblasts. Data indicate that Wnt3a up-regulates lysyl oxidase mRNA levels in progenitor cells while no effect was observed in either of the osteoblast models (Figure 1A). Treatment with purified recombinant Wnt3a showed similar results (Figure S1). Next, we assessed for lysyl oxidase transcriptional activity in response to Wnt3a using a firefly luciferase (pLOXFFL) reporter driven by the lysyl oxidase promoter. Analyses showed that Wnt3a up-regulates lysyl oxidase transcription activity in C3H10T1/2 cells (Figure 1B). Wnt3a also up-regulated pro-lysyl oxidase and mature lysyl oxidase protein levels in C3H10T1/2 cells (Figure 1C), and not in primary rat calvarial osteoblasts (Figure 1D). Data in Figure 1 support the conclusion that Wnt3a up-regulates lysyl oxidase mRNA levels at the transcriptional level in pluripotent mesenchymal progenitor cells, but not in committed osteoblasts.

### Wnt3a up-regulation of lysyl oxidase occurs through the Wnt canonical pathway

**Figure 2:**
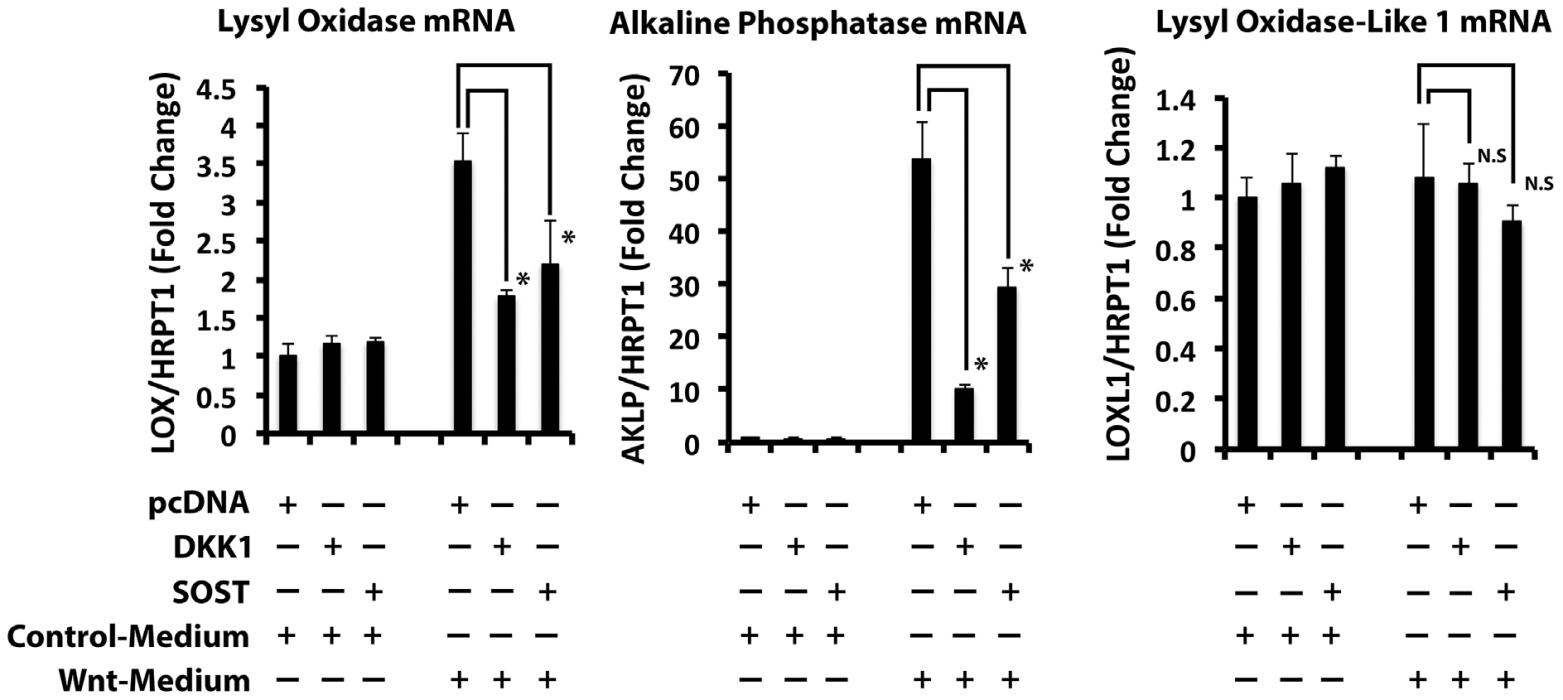
Wnt3a up-regulates lysyl oxidase through the canonical Wnt signaling pathway. DKK1 and SOST are inhibitors of the canonical Wnt pathway. DKK1 or SOST were over-expressed in C3H10T1/2 cells. The empty vector (pcDNA3.1^+^) used as a control. Transfected cells were serum starved prior to treatment with Wnt3a- or control conditioned medium for 24 hours. Total RNA was extracted and subjected to real time PCR analysis to measure mRNA levels of lysyl oxidase, alkaline phosphatase (AKLP), lysyl oxidase-like 1 (LOXL1). These mRNA levels were normalized to HPRT1 mRNA levels. Data are presented as means ± SD (n = 3; *, p<0.05, N.S, not significant; Student's t-test) and are representative of two independent experiments with the same outcome.

Wnt signaling can be mediated through the canonical or the non-canonical pathways [29]. Dickkopf-related Protein 1 (DKK1) and Sclerostin (SOST) each inhibit canonical Wnt signaling by binding to the Frizzled co-receptor LRP5/6 [30], [31]. The ability of these proteins to prevent Wnt3a-induced lysyl oxidase was evaluated by transfecting DKK1 or SOST expression vectors into C3H10T1/2 cells followed by treatment with Wnt3a- or control-conditioned medium for 24 hours. As a positive control, we assessed the effect of DKK1 and SOST on alkaline phosphatase (ALKP) mRNA levels [11]. To assess the specificity of Wnt3a, the mRNA levels of lysyl oxidase like 1 (LOXL1) in response to Wnt were also assessed. LOXL1 is the second most abundant protein of the lysyl oxidase family expressed by osteoblasts, after lysyl oxidase [32]. Data show that the over-expression of DKK1 and SOST in C3H10T1/2 cells strongly interfered with the Wnt3a induction of both ALKP and lysyl oxidase, but had no effect on LOXL1 mRNA levels (Figure 2). These data suggest that lysyl oxidase up-regulation by Wnt3a occurs through canonical Wnt signaling and is specific.

**Figure 3:**
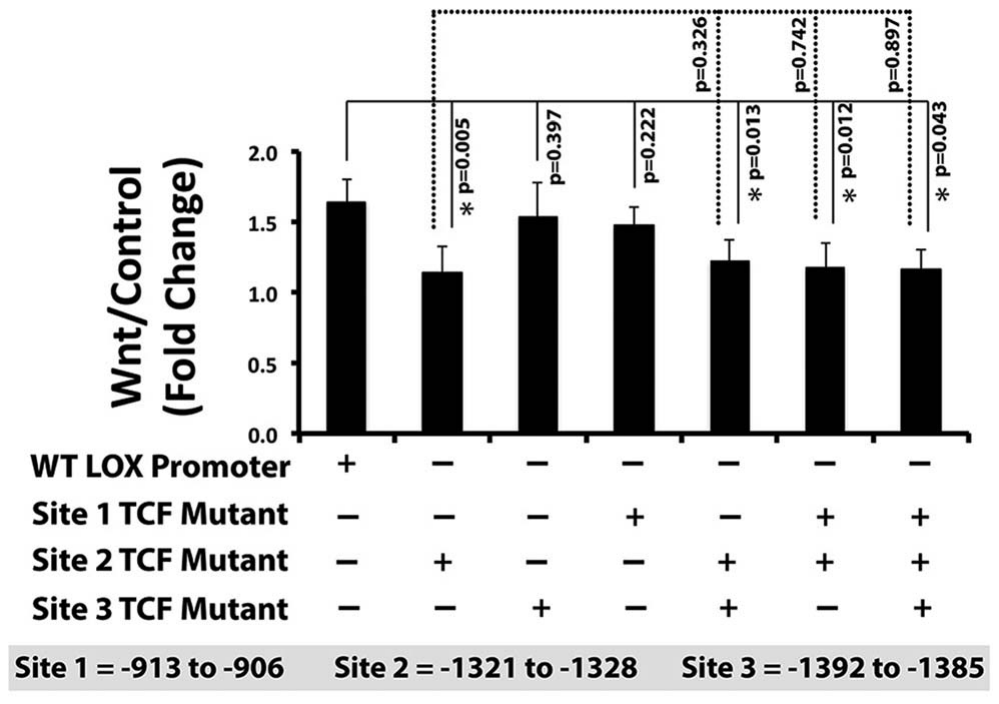
A potential cis-element for Wnt3a regulation of lysyl oxidase is located at −1321 to −1328 bp upstream of lysyl oxidase translation start site. Three putative TCF/LEF cis-elements within the first 1.5 kbp of the murine lysyl oxidase promoter (pLOXFL) were mutated by site-directed mutagenesis individually, in pairs, and all three together. C3H10T1/2 cells were transfected with a Renilla luciferase thymidine kinase (pRL-TK) and either wild type pLOXFFL or mutant pLOXFFL reporter constructs. After 24 hours, transfected cells were serum starved and treated with Wnt3a- or control-conditioned media for 24 hours. Luciferase activity was assessed as explained in Experimental Procedures. The fold change of lysyl oxidase transcriptional activity of wild-type and mutated reporter constructs in response to Wn3a are presented as means ± SD. Data are pooled from three independent experiments (n = 9; *, p<0.05; Student's t-test).

To further establish the transcriptional regulation of lysyl oxidase by the canonical Wnt pathway, we next mutated three putative TCF/LEF cis-elements individually, in pairs, and all three together (Experimental Procedures and Table 3). Data in Figure 3 indicate that mutation of the putative TCF/LEF site at −1321 to −1328 upstream of the translation start site inhibited the up-regulation of lysyl oxidase transcriptional activity by Wnt3a in C3H10T1/2 cells. This finding was confirmed in all double and triple mutants containing mutations in the −1321 to −1328 site (site 2) but not in constructs containing mutations in site 1 or site 3 that lacked the site 2 mutation (Figure 3). Collectively, these experiments strongly support that Wnt3a up-regulates lysyl oxidase transcription through the canonical Wnt signaling pathway. This is the first identification of a putative functional TCF/LEF cis-acting transcriptional element in the lysyl oxidase promoter.

### TNF-α down-regulates lysyl oxidase in Wnt3a-stimulated and non-stimulated C3H10T1/2 cells

**Figure 4:**
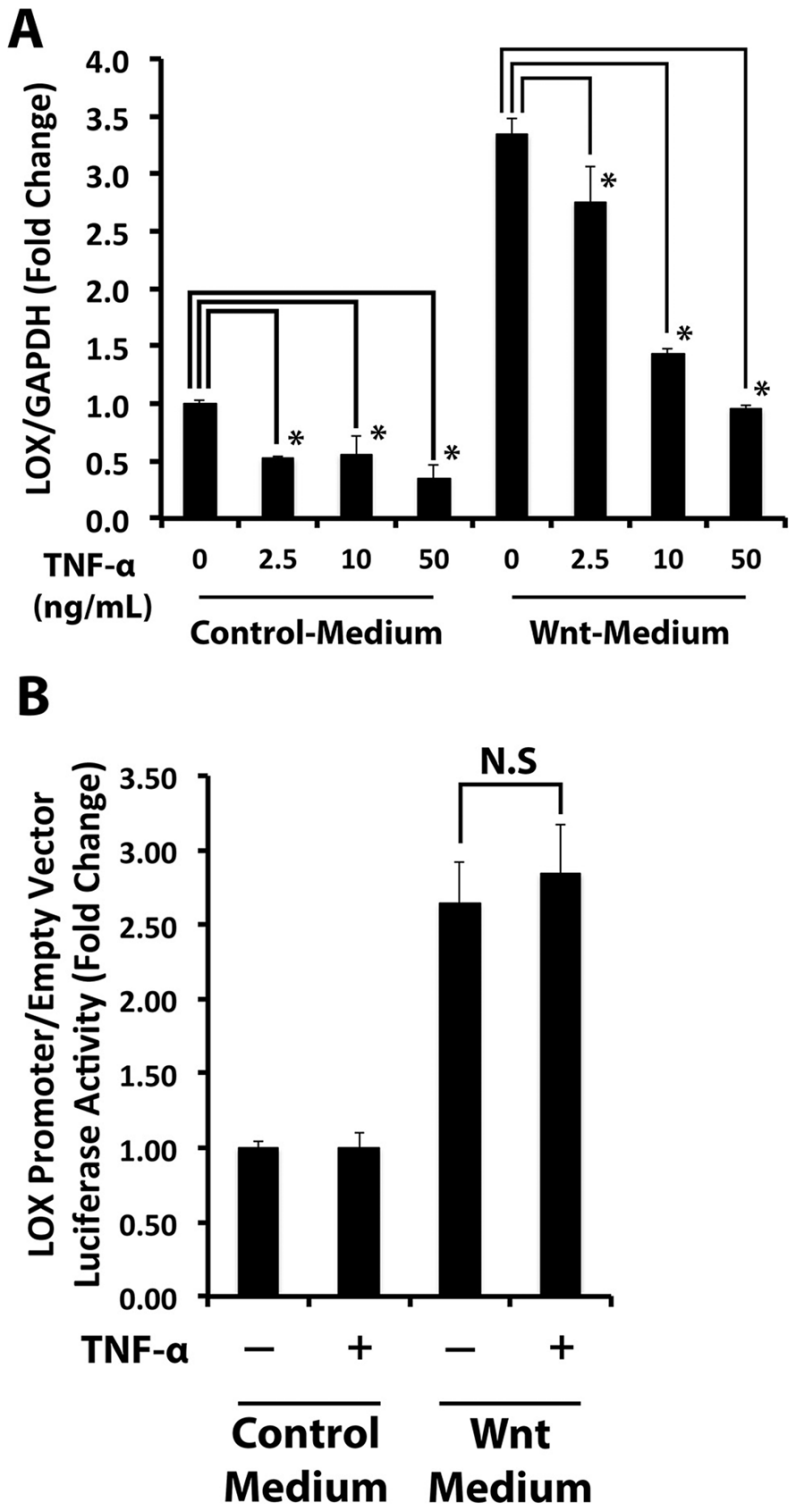
TNF-α post-transcriptionally attenuates Wnt3a-stimulated lysyl oxidase mRNA levels. **A)** The effect of TNF-α on Wnt3a-stimulated lysyl oxidase mRNA levels in C3H10T1/2 cells was examined by treating cells with Wnt3a- or control-conditioned media in the presence or absence of increasing concentrations of TNF-α. Lysyl oxidase mRNA analysis of total RNA extracted from these cells was performed by real time PCR. Lysyl oxidase mRNA levels were normalized to the levels of GAPDH mRNA. Data are means ± SD (n = 3; *, p<0.05; Student's t-test). **B)** Luciferase reporter was used to functionally assess lysyl oxidase transcriptional activity in response to TNF-α. Data are means ± SD (n = 6, N.S, not significant; Student's t-test). Data shown were pooled from two independent experiments.

Elevated levels of TNF-α have been linked to bone pathologies including diabetic osteopenia [33]. TNF-α stimulates FOXO-dependent transcription which can divert β-catenin away from binding to TCF/LEF cis-acting promoter elements [34]. We therefore wished to determine whether TNF-α would interfere with Wnt3a-dependent regulation of lysyl oxidase mRNA levels at this transcriptional level. Serum-depleted C3H10T1/2 cells were treated with Wnt3a- or control-conditioned medium in the presence or absence of various concentrations of TNF-α for 24 hours. Analyses of RNA by qPCR indicate that TNF-α reduced Wnt3a-stimulated lysyl oxidase mRNA levels and this effect was dose-dependent (Figure 4A). Down-regulation of basal lysyl oxidase mRNA by TNF-α was also observed (Figure 4A). To determine whether this TNF-α effect occurred at the level of transcription, C3H10T1/2 cells were transfected with pLOXFFL and pRL-TK, serum starved and then treated with Wnt3a- and control-conditioned medium in the presence or absence of 20 ng/ml TNF-α for 24 hours. Intriguingly, TNF-α did not impair basal or Wnt-3a-stimulated lysyl oxidase transcriptional activity (Figure 4B), by contrast to its down-regulation of Wnt-3a-stimulated lysyl oxidase steady state mRNA levels. Moreover, TNF-α treatment did not interfere with Wnt canonical signaling determined by the pTOPFLASH/pFOPFLASH reporter assay which independently assesses for TCF/LEF-dependent transcriptional activation (Figure S2). This suggests that TNF-α inhibition of Wnt3a-stimulated lysyl oxidase mRNA is likely mediated by post-transcriptional mechanisms.

**Figure 5:**
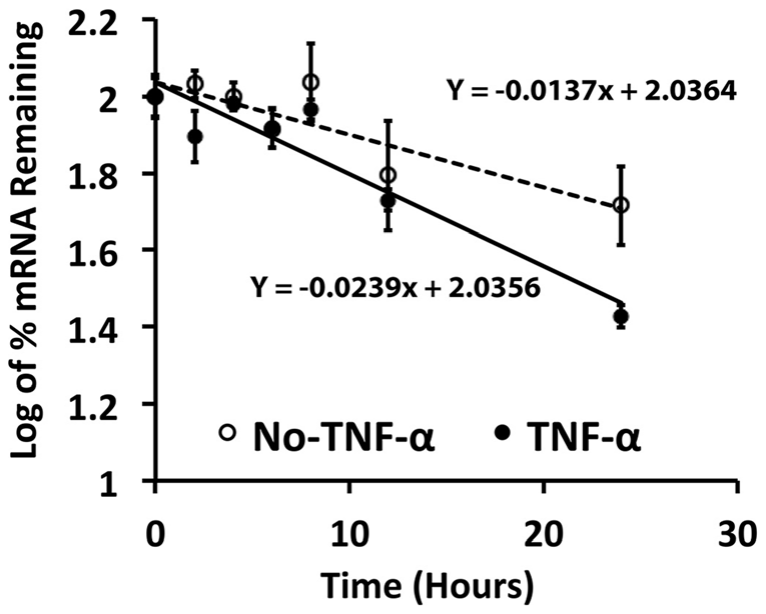
TNF-α reduces Wnt3a-stimulated lysyl oxidase mRNA stability. Serum starved C3H10T1/2 were pre-treated with Wnt3a- or conditioned media for 16 hours and then treated with or without TNF-α (20 ng/ml) in the presence of dichlorobenzimidazole riboside (an inhibitor of mRNA transcription) for various intervals. Total RNA was isolated from cell lysates and subjected to real time PCR. Data were plotted as percent log remaining lysyl oxidase mRNA levels (normalized to 18 s rRNA mRNA levels) vs time. The TNF-α induced loss of lysyl oxidase mRNA stability was calculated from the relative slopes of the lines of best fit. Student's t-test statistical analyses were performed by comparing the slopes and intercepts of these two lines and showed a P value of 0.006 (GraphPad Prism 5). Data are means ± SD (n = 3 for each time point) and are from one of two representative experiments with the same outcome.

Next, we explored the possibility that TNF-α acted to decrease lysyl oxidase mRNA stability. Serum-depleted C3H10T1/2 cells were pre-treated with Wnt3a-conditioned medium for 16 hours followed by TNF-α (20 ng/ml) or vehicle for 4 hours. Cells were then supplemented with 20 µg/ml of dichlorobenzimidazole riboside (DRB) to inhibit RNA polymerase II and mRNA transcription. Total RNA was harvested at intervals and lysyl oxidase mRNA levels were measured by real time PCR. The data (Figure 5) show a 50% reduction in the stability of Wnt3a-stimulated lysyl oxidase mRNA in response to TNF-α.

### TNF-α down-regulates lysyl oxidase via miRNAs

We hypothesized that the down-regulation of Wnt3a-stimulated lysyl oxidase mRNA levels by TNF-α might be due to its induction of a microRNA that targets lysyl oxidase mRNA. The miRNome microRNA PCR array was used to screen for microRNAs that were up-regulated by TNF-α in Wnt3a-stimulated C3H10T1/2 cells. Serum-depleted cells were stimulated with Wnt3a-conditioned medium and then treated with or without TNF-α (20 ng/mL). Total RNA, including microRNA, was subjected to a microRNA PCR array analysis (Experimental Procedures). A scatter plot of the log of each normalized microRNA level in TNF-α treated versus control non-TNF-α treated cells is shown in Figure S3 in order to view microRNAs which were differentially expressed using a threshold cutoff of 4-fold. Data identify 39 differentially expressed microRNAs out of 440 assayed. Only ten of these 39 microRNAs were up-regulated by TNF-α and these are listed in Table 4. In silico analysis using the miRanda algorithm [35] next identified one of the ten up-regulated microRNAs (miR203) as having the potential to target mouse lysyl oxidase mRNA.

**Table 4:** Micro RNAs Increased More Than 4-fold by TNF-α in Wnt3a-stimulated C3H10T1/2 Cells.

MicroRNA ID	TNF-α: Non-TNF-α (Fold Change)
miR-1187	946.6365
miR-146a	47.5048
miR-155	13.1775
**miR-203**	**13.147**
miR-208a	11.2096
miR-202-3p	5.7624
miR-450b-3p	5.2054
miR-1190	4.9474
miR-1938	4.5211
miR-196a	4.1892

**Figure 6:**
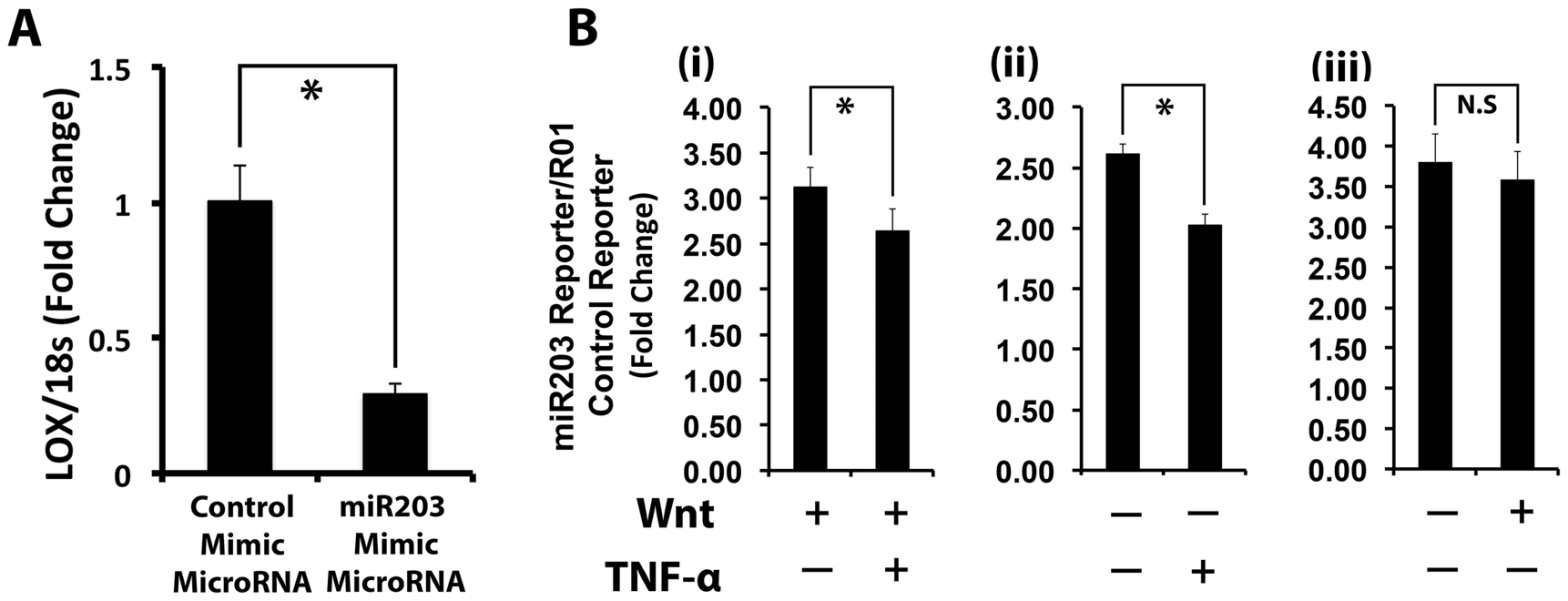
Analysis of miR203 functionality to down-regulate lysyl oxidase mRNA levels. **A)** miR203 down-regulates lysyl oxidase mRNA levels: C3H10T1/2 cells were transfected with miR203 mimic or non-specific control micro RNA. Cell lysates were collected after five days post-transfection and total extracted RNA subjected to real time PCR analysis for lysyl oxidase and 18S rRNA mRNA for normalization. Data are presented as means ± SD (n = 3; *, p<0.05; Student's t-test). Data are from one of two independent experiments with the same outcome. **B**) To functionally evaluate TNF-α up-regulation of functional miR203 in C3H10T1/2 cells, cells were transfected with the miR203 reporter plasmid miR203-RenSPL and R01-RenSPL (control) constructs. 24 Hours post-transfection, cells were serum starved and pre-treated with (i) Wnt3a- or (ii) control conditioned media for 24 hours. Cells were then treated with or without TNF-α (20 ng/ml) for 24 hours. Cell lysates were collected and subjected to a luciferase activity assay. Panel (iii) is from cells treated only with or without Wnt3a showing that this reporter for miR203 activity is not affected by Wmt3a alone, as expected. Data are presented as means ± SD (n = 6; *, p<0.05; N.S, not significant; Student's t-test). Data shown are from one of two independent experiments with the same outcomes.

We next investigated whether miR203 directly down-regulates lysyl oxidase mRNA levels. C3H10T1/2 cells were transfected with a miR203 mimic or control microRNA (AllStars siRNA). miR203 mimic is a synthetic double stranded RNA, and can be directly transfected into cells. The mature miR203 sequence is 5′UGAAAUGUUUAGGACCACUAG. Total RNA was collected from cell layers five days post-transfection and lysyl oxidase mRNA levels were assessed by real time PCR. Lysyl oxidase mRNA levels were reduced down to 20% of control levels in cells transfected with the miR203 mimic (Figure 6A). We next functionally verified the up-regulation of miR203 in Wnt3a-stimulated C3H10T1/2 in response to TNF-α in the presence or absence of Wnt3a pretreatment using a miR203 luciferase reporter (miR203-RenSPL) which generates an mRNA that can be targeted by a functional and fully processed miR203. The luciferase activity of miR203 reporter is inversely correlated with the levels of fully processed and functional miR203 because the transcript of miR203-RenSP construct has a specific binding site for miR203. Thus, cells were transfected with miR203 or R01 (control) luciferase, starved overnight, and treated with Wnt3a- or control-conditioned media for 24 hours. Cells were next treated with or without TNF-α (20 ng/ml) for an additional 24 hours, and luciferase activity was determined in cell lysates. The results revealed that TNF-α induces miR203 in C3H10T1/2 cells under both Wnt3a-stimulated (Figure 6B, panel i) and non-stimulated conditions (Figure 6B, panel ii, evident as reduced luciferase activity in these groups. Figure 6B panel iii shows that Wnt3a treatment does not affect the activity of this miR203 reporter, as expected. Taken together, the results from these two experiments confirmed that miR203 is induced by TNF-α and reduces lysyl oxidase mRNA levels in a model of pluripotent mesenchymal cells.

### Biological role for lysyl oxidase in C3H10T1/2 cells

**Figure 7:**
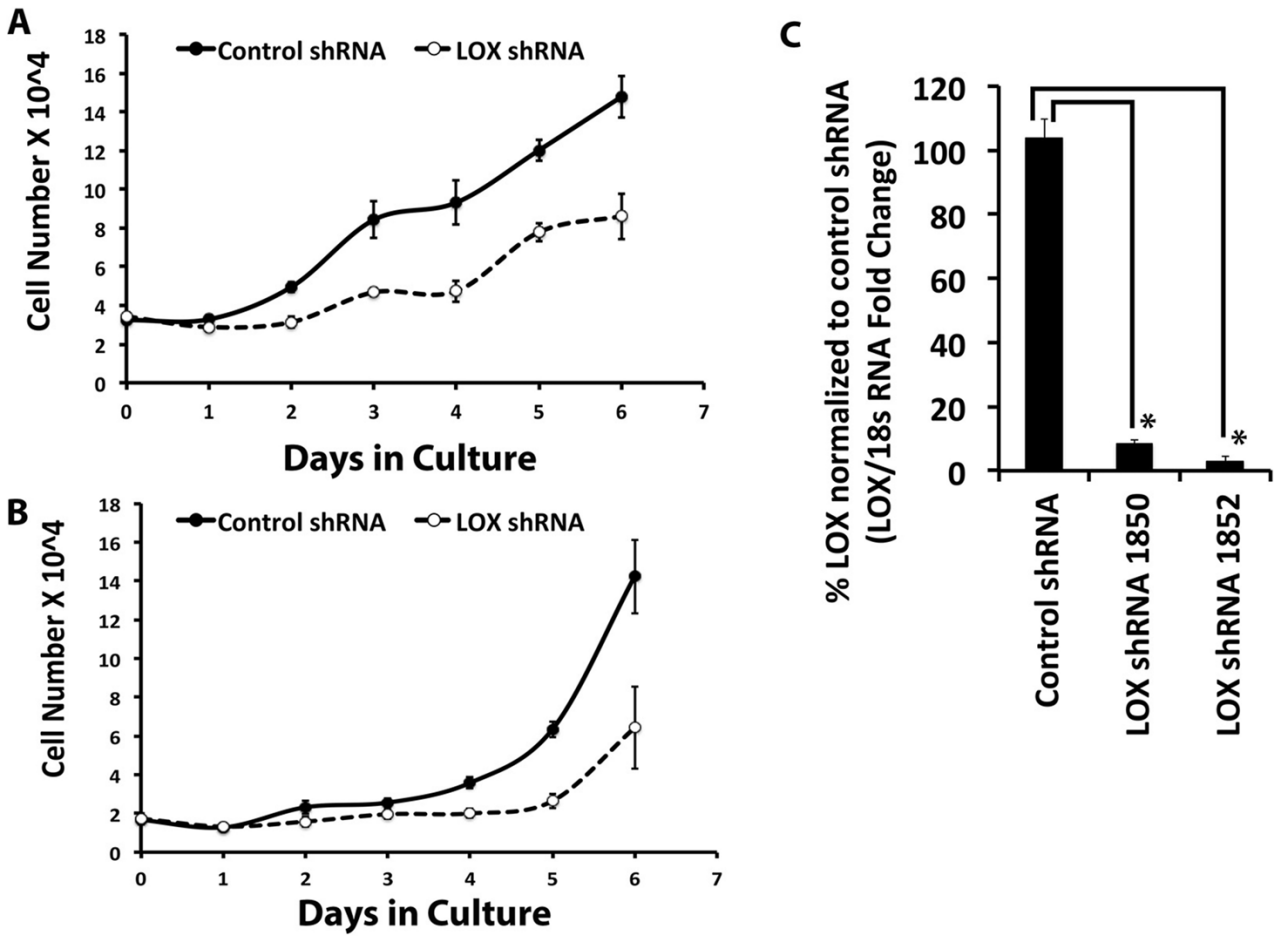
Lysyl oxidase (LOX) regulates C3H10T1/2 cell growth. C3H10T1/2 cells were transduced with lentiviral particles containing LOX shRNA1850, LOX shRNA 1852, or control shRNA. Non-transduced cells were eliminated using puromycin, a selective reagent. Transduced cells were seeded at 20,000 cells per well in 12-well plates for the growth curve analysis. Total RNA was also collected from these cells to measure lysyl oxidase mRNA levels by real-time PCR. **A)** The growth curves were plotted for cells transduced with either LOX shRNA 1852 or control shRNA. Data are presented as means ± SD (n = 4). **B)** The growth curves were plotted for cells transduced with either LOX shRNA 1850 or control shRNA. Data are presented as means ± SD (n = 4). **C)** The chart shows lysyl oxidase mRNA levels in LOX knockdown and control cells. Two independent LOX shRNA with different shRNA sequences were used in this experiment. Data are presented as means ± SD (n = 3*, p<0.05; Student's t-test).

**Figure 8:**
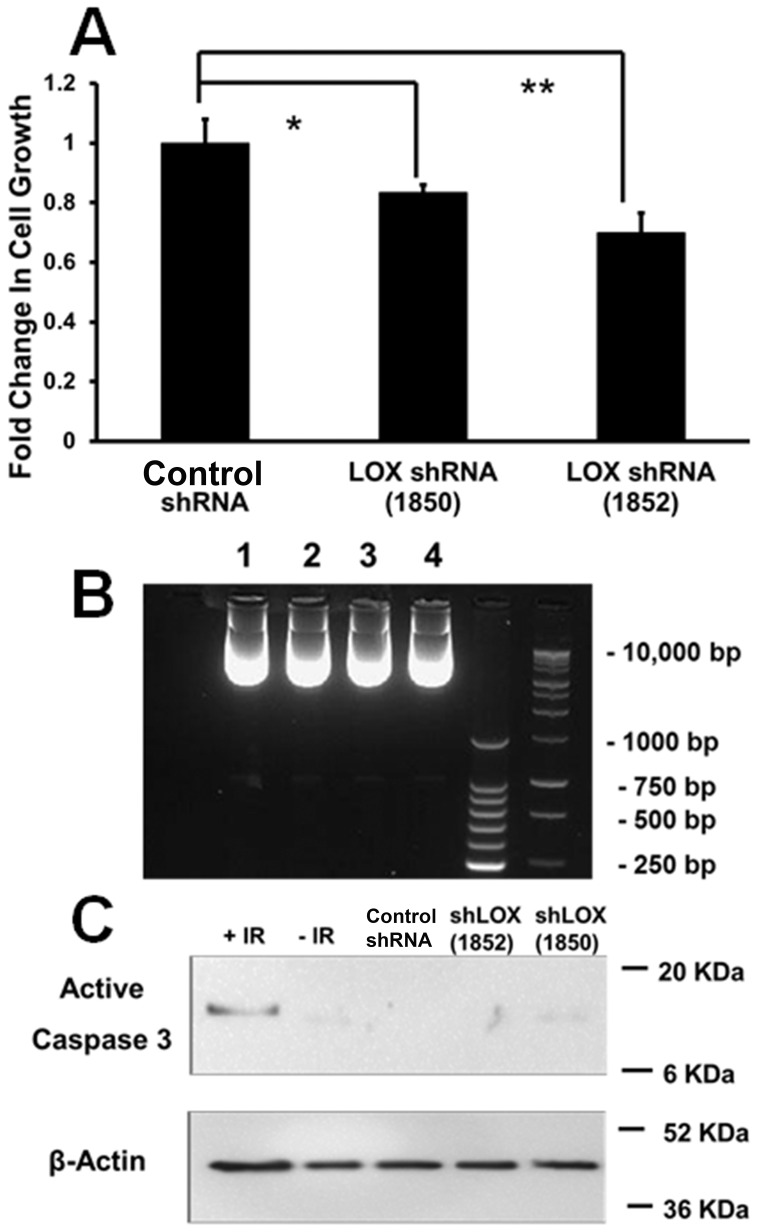
LOX shRNA inhibits C3H10T1/2 cell proliferation and does not stimulate cell death. (**A**) DNA accumulation over a 24-hour period as an index of proliferation was determined in sub-confluent C3H10T1/2 cells as described in Materials and Methods. Data shown are means +/− SD; n = 4. (B) DNA fragmentation was assayed after isolation of genomic DNA and 2% agarose gel electrophoresis stained with ethidium bromide. 24 Micrograms of DNA was loaded per sample. Lane 1, scrambled shRNA; lane 2, LOX shRNA 1850; lane 3, LOX shRNA 1852, lane 4, non-transduced C3H10T1/2 cells. This experiment was performed 3 times with the same outcome. (C) Western blot of cell extracts for active caspase-3 shows no difference as a function of LOX knockdown, while the positive control of C3H10T1/2 cells subjected to 20 Gy radiation exhibited activated caspase 3 as expected.

C3H10T1/2 pluripotent cells are non-differentiated, and not highly active in the production of the extracellular matrix, while osteoblasts produce abundant amounts of cross-linked collagen. Our expectation was that osteoblasts which are known to be responsive to Wnt3a [12] would exhibit lysyl oxidase regulation in response to Wnt3a. As noted, data instead show that C3H10T1/2 cells exhibited increased lysyl oxidase levels in response to Wnt3a, while osteoblasts did not (Figure 1). We, therefore, next asked what function lysyl oxidase could have in the pluripotent cells and what the significance of Wnt3a stimulated lysyl oxidase levels could be. As noted, Wnt3a was reported to be mitogenic for pre-osteoblasts [9]. The degree to which lysyl oxidase expression contributes to proliferation and accumulation of these cells was investigated. Lysyl oxidase expression was knocked down in C3H10T1/2 cells, respectively using two different shRNAs which target different sequences of lysyl oxidase, and growth curves were analyzed as described in Materials and Methods. Data in Figure 7A and 7B show that each lysyl oxidase-directed shRNA resulted in potent inhibition of growth compared to control shRNA transduced C3H10T1/2 cells. Data in Figure 7C confirm that the shRNAs knocked down lysyl oxidase mRNA and protein levels (Figure S4). To further investigate changes in the balance between proliferation and cell death in C3H10T1/2 cells in which lysyl oxidase expression was knocked-down, short-term DNA accumulation assays for proliferatve activity, and DNA fragmentation and caspase assays for cell death were performed. 24-Hour DNA accumulation was measured using the CyQuant assay as described in Materials and Methods, and confirmed that DNA accumulation was inhibited by each of the two lysyl oxidase shRNAs employed (Figure 8A). Figure 8B and 8C respectively show no evidence for increased DNA fragmentation or increased caspase 3 activation as a function of lysyl oxidase knockdown. Taken together data indicate that lysyl oxidase functions to maintain the ability of pluripotent cells to proliferate.

**Figure 9:**
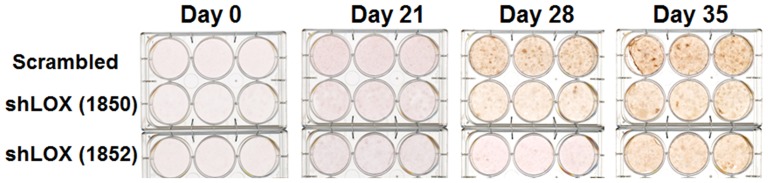
Lysyl oxidase knockdown prevents osteoblast development of C3H10T1/2 cells. C3H10T1/2 cells transduced with two different lysyl oxidase shRNAs or empty virus were grown to confluence and then induced to differentiate as indicated in Materials and Methods. At intervals, cells were fixed and stained with alizarin red. Fixed and stained cultures were then photographed. This experiment was performed twice with the same outcomes.

To further assess for the functionality of lysyl oxidase in C3H10T1/2 cells, lysyl oxidase shRNA and control transduced cultures were grown under conditions which promote osteoblast differentiation, and were stained at intervals with alizarin red. Data in Figure 9 indicate that differentiation was inhibited in lysyl oxidase shRNA transduced cells. Thus, development of osteoblasts from pluripotent mesenchymal cells requires lysyl oxidase expression.

## Discussion

An important complication of diabetes is diabetic bone disease or diabetic osteopenia [36]. Inflammation generally and TNF-α in particular is elevated in inflammatory conditions including diabetes and contributes to diabetic complications in mineralized tissues [33]. TNF-α signaling initiates formation of intermediates including increased levels of reactive oxygen species, which can inhibit aspects of canonical Wnt signaling in osteoblasts [12]. Wnt signaling has been implicated as a key regulator of bone development and homeostasis [10]. In order to gain new insights into effects of inflammation on bone homeostasis, we investigated possible relationships between Wnt3a signaling and effects of TNF-α on lysyl oxidase expression in models of non-differentiated mesenchymal cells, and in committed pre-osteoblasts.

The function of lysyl oxidase in a pluripotent cell that makes little or no extracellular matrix was not immediately obvious. Investigations into a potential role for lysyl oxidase in promoting or permitting cell growth resulted in the discovery of a very significant inhibition of cell growth using two different shRNA knockdown constructs for lysyl oxidase. This finding and the lack of Wnt3a effects on osteoblasts to regulate lysyl oxidase are unexpected because lysyl oxidase function is classically considered to be dedicated to the maturation of collagens in the context of bone formation. The mechanisms by which lysyl oxidase promotes cell proliferation may involve some of its reported activities to enzymatically modify growth factor receptors, growth factors, or non-enzymatic functions mediated by the biologically active lysyl oxidase propeptide. Among these possibilities is the ability of lysyl oxidase to activate PDGF receptor signaling which could be a consequence of its direct modification of a PDGF receptor [37]. Lysyl oxidase enzyme has also been implicated in activating FAK signaling, though the functional substrates in this context have not been identified [38], [39]. Lysyl oxidase has been reported to oxidize and inactivate the functions of FGF-2 and TGF-β by direct enzymatic modification [40], [41]. Although FGF-2 and TGF-β are each mitogenic for mesenchymal cells, oxidation and inactivation of posttranslational activators of latent TGF-β or of FGF co-receptors could lead to a positive role for lysyl oxidase in stimulating cell proliferation [42], [43]. These potential mechanisms of action are under investigation.

An important biological implication of this work is that lysyl oxidase is needed for an adequate supply and reservoir of pluripotent cells for mineralized tissue synthesis and maintenance. Excess TNF-α levels in inflammatory diseases including diabetes may contribute to osteopenia by inhibiting the proliferation of pluripotent cells by down-regulating lysyl oxidase. Interestingly, lysyl oxidase expression is also required for C3H10T1/2 cell differentiation into adipocytes [20]. We suggest that a role for lysyl oxidase in pluripotent cells is to help provide an adequate supply of cells which can subsequently differentiate into adipocytes, or alternatively into chondrocytes or osteoblasts.

In summary, data indicate that Wnt3a stimulates lysyl oxidase expression only in C3H10T1/2 cells and in primary bone marrow pluripotent cells, but not in committed osteoblasts. TNF-α was found to inhibit lysyl oxidase expression, but not through the expected transcriptional mechanism, but rather through miR203. A function for lysyl oxidase in promoting the proliferation of C3H10T1/2 cells was found. This activity of lysyl oxidase which is respectively targeted positively or negatively by Wnt3a and TNF-α may be important in maintaining an adequate supply of pluripotent cells that can be recruited for differentiation into mature functional extracellular matrix producing connective tissue and cells, or alternatively adipose tissue.

## Supporting Information

Figure S1 **Recombinant Wnt3a up-regulates lysyl oxidase in C3H10T1/2 pluripotent progenitor cells.** C3H10T1/2 and MC3T3 cells were serum depleted overnight and treated with recombinant Wnt3a (150 ng/ml) for 24 hours. Real time PCR analysis of total RNA indicates that Wnt3a up-regulates lysyl oxidase mRNA levels in C3H10T1/2 cells while it fails to induce lysyl oxidase in MC3T3 pre-osteoblasts. Data are presented as means ± SD and are from one of two independent experiments with the same outcomes (n = 3; *, p<0.05, N.S, not significant; Student's t-test).
(TIFF)

Figure S2 **TNF-α does not interfere with the canonical Wnt signaling.** The pTOPFLASH and pFOPFLASH reporters were used to assess for TNF-α regulation of canonical Wnt signaling activity. C3H10T1/2 cells were transfected with Renilla luciferase thymidine kinase (pRL-TK). and either pTOPFLASH and control pFOPFLASH reporters. Cells were then treated with Wnt3a- or control-conditioned media supplemented with or without TNF-α (20 ng/ml) for 24 hours. The reporter activities in response to Wnt3a and TNF-α with Wnt3a were plotted. Data are presented as means ± SD (n = 3; *, p<0.05, N.S, not significant). Data are from one of two independent experiments with the same outcomes.
(TIFF)

Figure S3 **TNF-α up-regulates miR203 in Wnt3a-stimulated pluripotent progenitor cells.** Serum starved C3H10T1/2 were pre-treated with Wnt3a-conditioned medium for 16 hours and then treated with or without TNF-α (20 ng/ml) for 24 hours. We then profiled 440 mouse micro RNAs using a micro RNA PCR array analysis as indicated in Experimental Procedures. The scatter plot shows the log of the probed normalized microRNAs levels in TNF-α treated and non-TNF-α treated cells. The outer lines (red) mark the 4-fold threshold difference of microRNA ratios between TNF-α treated and non-TNF-α treated cells.
(TIF)

Figure S4 **Lysyl oxidase protein knockdown in C3H10T1/2 cells.** The LOX shRNA was used to knockdown lysyl oxidase protein levels in C3H10T1/2 cells. Cells were transduced with lentiviral particles containing LOX shRNA or control shRNA. Cell lysates were then were subjected to Western blotting. The chart shows lysyl oxidase protein levels for LOX knockdown and control C3H10T1/2 cells. Data are presented as means ± SD (n = 3; *, p<0.05).
(TIF)
