# Supplementary figures and images for: A Novel Function for Lysyl Oxidase in Pluripotent Mesenchymal Cell Proliferation and Relevance to Inflammation-Associated Osteopenia
Source: PLoS One. 2014 Jun 27;9(6):e100669. doi: 10.1371/journal.pone.0100669 (PMC4074096; doi:10.1371/journal.pone.0100669)

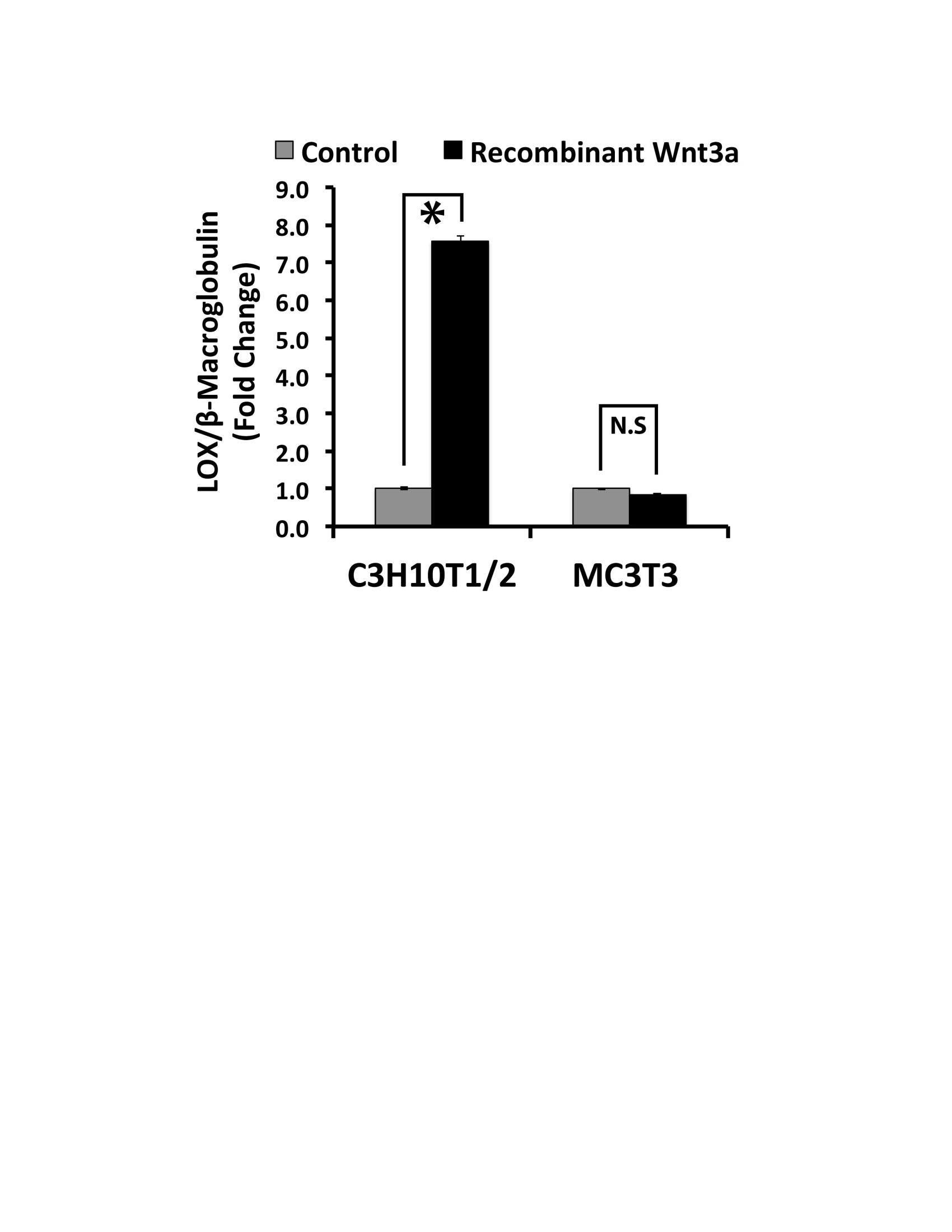

Supplement: Figure S1 — Recombinant Wnt3a up-regulates lysyl oxidase in C3H10T1/2 pluripotent progenitor cells. C3H10T1/2 and MC3T3 cells were serum depleted overnight and treated with recombinant Wnt3a (150 ng/ml) for 24 hours. Real time PCR analysis of total RNA indicates that Wnt3a up-regulates lysyl oxidase mRNA levels in C3H10T1/2 cells while it fails to induce lysyl oxidase in MC3T3 pre-osteoblasts. Data are presented as means ± SD and are from one of two independent experiments with the same outcomes (n = 3; *, p<0.05, N.S, not significant; Student's t-test). (TIFF) [file pone.0100669.s001.tiff]

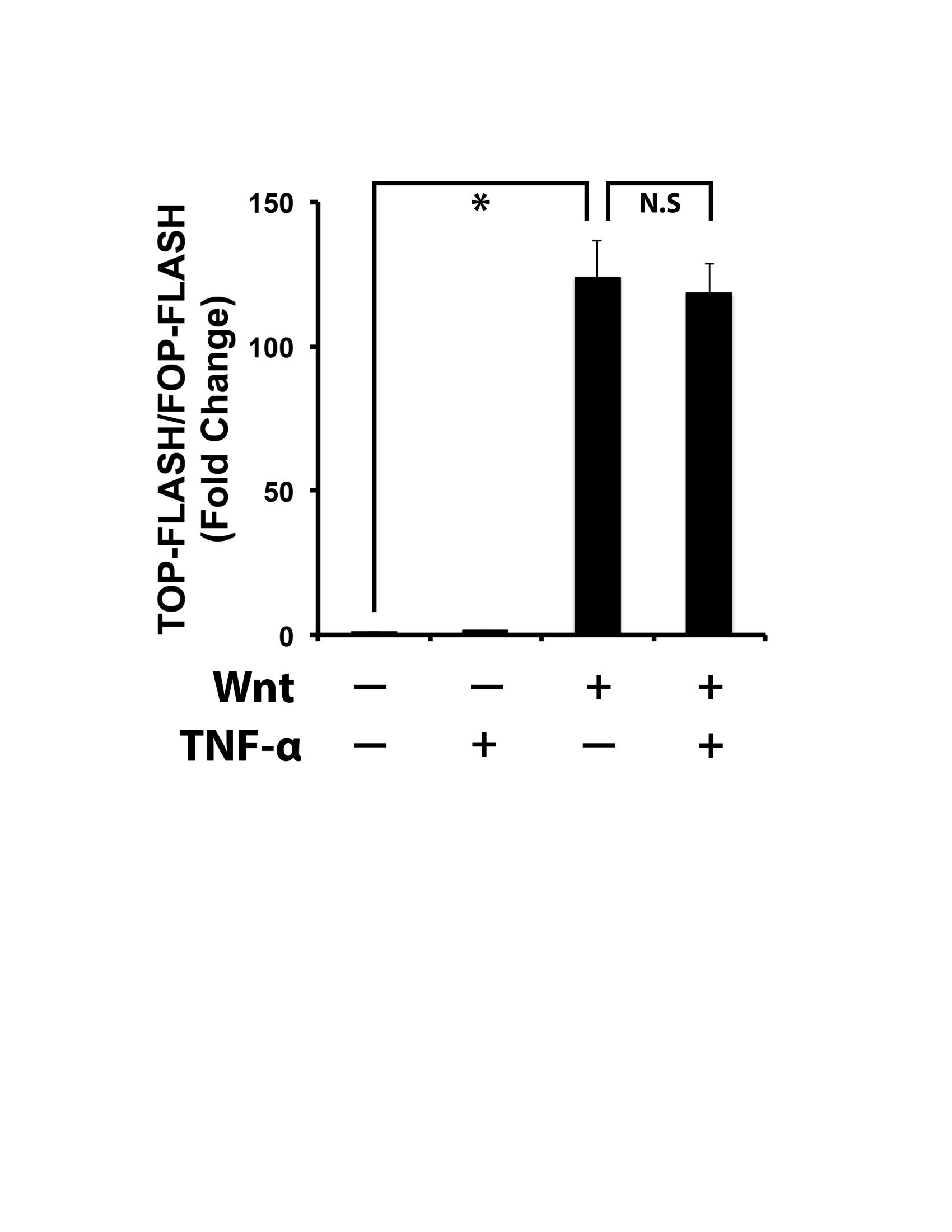

Supplement: Figure S2 — TNF-α does not interfere with the canonical Wnt signaling. The pTOPFLASH and pFOPFLASH reporters were used to assess for TNF-α regulation of canonical Wnt signaling activity. C3H10T1/2 cells were transfected with Renilla luciferase thymidine kinase (pRL-TK). and either pTOPFLASH and control pFOPFLASH reporters. Cells were then treated with Wnt3a- or control-conditioned media supplemented with or without TNF-α (20 ng/ml) for 24 hours. The reporter activities in response to Wnt3a and TNF-α with Wnt3a were plotted. Data are presented as means ± SD (n = 3; *, p<0.05, N.S, not significant). Data are from one of two independent experiments with the same outcomes. (TIFF) [file pone.0100669.s002.tiff]

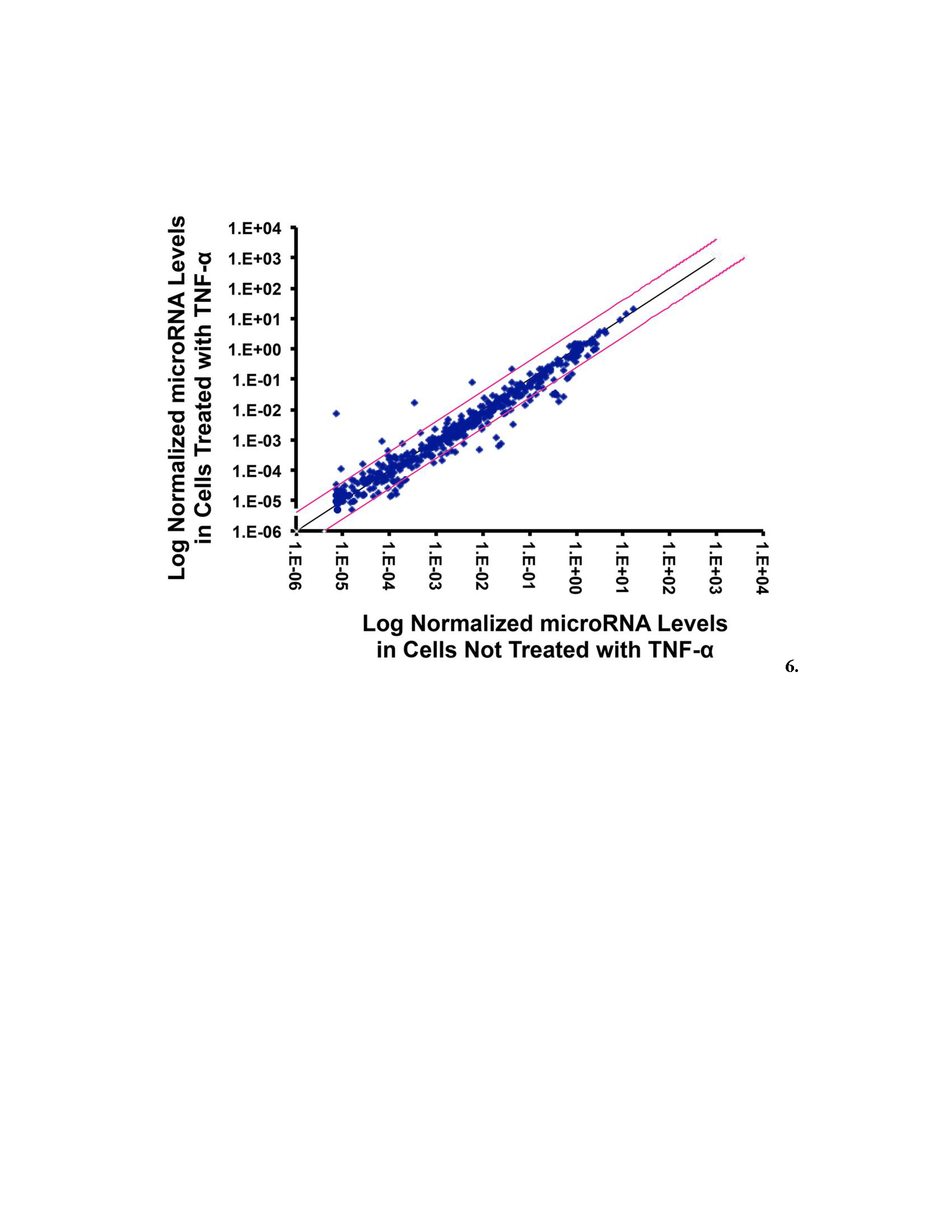

Supplement: Figure S3 — TNF-α up-regulates miR203 in Wnt3a-stimulated pluripotent progenitor cells. Serum starved C3H10T1/2 were pre-treated with Wnt3a-conditioned medium for 16 hours and then treated with or without TNF-α (20 ng/ml) for 24 hours. We then profiled 440 mouse micro RNAs using a micro RNA PCR array analysis as indicated in Experimental Procedures. The scatter plot shows the log of the probed normalized microRNAs levels in TNF-α treated and non-TNF-α treated cells. The outer lines (red) mark the 4-fold threshold difference of microRNA ratios between TNF-α treated and non-TNF-α treated cells. (TIF) [file pone.0100669.s003.tif]

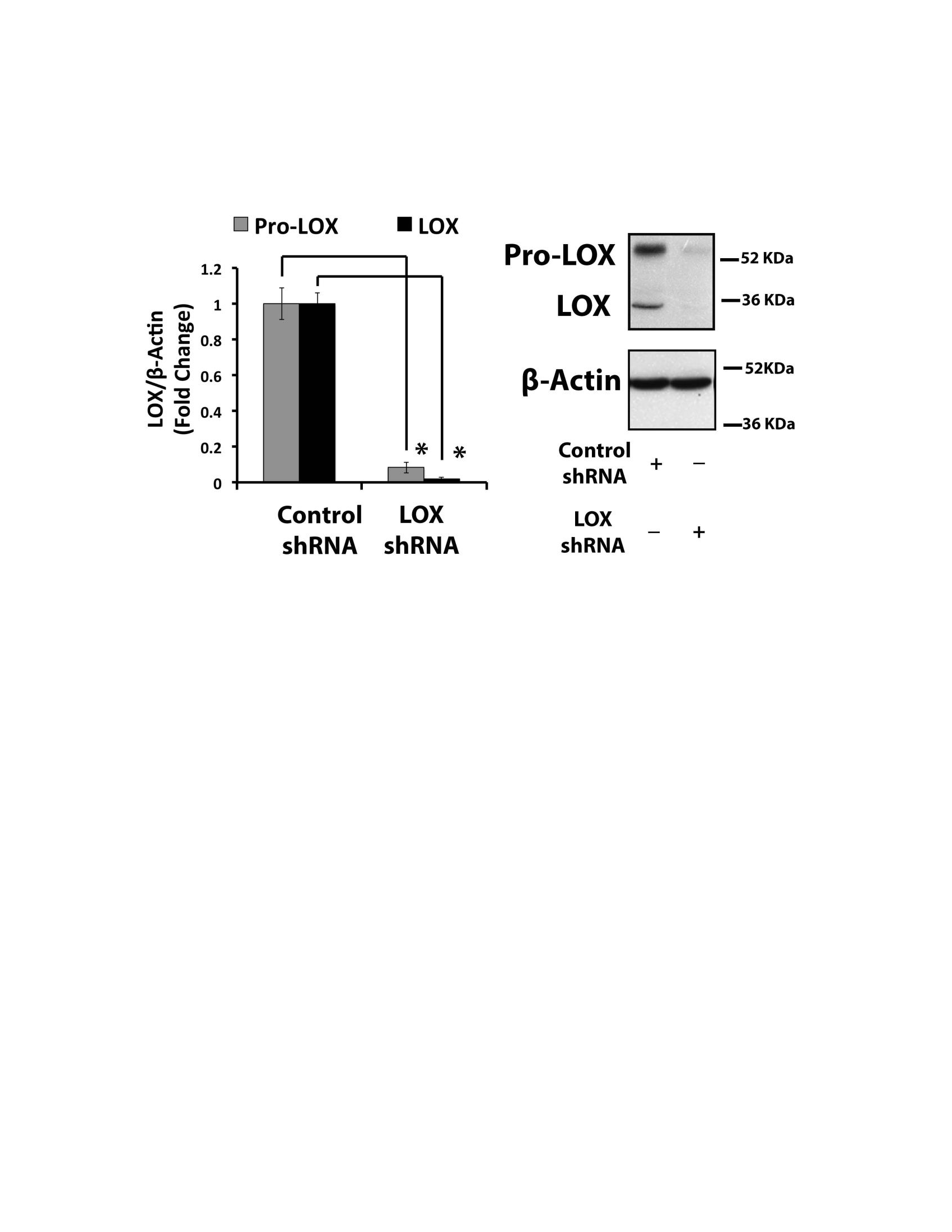

Supplement: Figure S4 — Lysyl oxidase protein knockdown in C3H10T1/2 cells. The LOX shRNA was used to knockdown lysyl oxidase protein levels in C3H10T1/2 cells. Cells were transduced with lentiviral particles containing LOX shRNA or control shRNA. Cell lysates were then were subjected to Western blotting. The chart shows lysyl oxidase protein levels for LOX knockdown and control C3H10T1/2 cells. Data are presented as means ± SD (n = 3; *, p<0.05). (TIF) [file pone.0100669.s004.tif]
